# Fifty top-cited fracture articles from China: a systematic review and bibliometric analysis

**DOI:** 10.1186/s13018-016-0408-8

**Published:** 2016-07-01

**Authors:** Fang Dong, Mengpo Fan, Zhiwei Jia

**Affiliations:** Department of Orthopaedics, The Second People’s Hospital of Liaocheng, Shandong, China; Department of Orthopaedics, The 306th Hospital of People’s Liberation Army, Beijing, China

**Keywords:** Fracture surgery, Bibliometric analysis

## Abstract

**Background:**

With more than 50,000 orthopaedic surgeons, China is having an increasing impact on fracture surgery research. However, the most influential Chinese articles on fracture surgery have not been determined. This study aimed to characterise the most-cited articles on fracture surgery by Chinese authors to provide insight into the fracture research in China.

**Methods:**

The Web of Science was used to search for citations of fracture surgery articles that originated in China. The 50 most-cited articles were identified. The title, number of citations, year of publication, journal, article type, level of evidence, city, institution, and authors were recorded and evaluated.

**Results:**

The 50 top-cited papers were published between 1984 and 2012. The most prolific decade began in the year 2000. These articles received 28 to 209 citations (mean 52), were written in English, and published in 12 journals. *Injury* was the most popular journal, with the largest number of articles (11) on the top 50 list. The region with the largest number of published articles was Hong Kong (20), followed by Kaohsiung (8), Shanghai (8), and Taipei (7). Most were clinical studies (39), while the remaining studies were basic science articles (11). The hip was the most common topic in the clinical studies. The most popular level of evidence was IV.

**Conclusions:**

This list of the top 50 publications identifies the most influential Chinese fracture surgery articles for the global community. This study presents insight into the historical contributions of Chinese researchers and the fracture surgery trends in China.

## Background

The number of citations that a paper receives is a significant indicator of its influence and contribution to a certain field [1–3]. Citation analysis is an important measurement for assessing the academic significance of a paper [1–3]; it is a bibliometric method used to investigate the characteristics of a paper’s citations. Many bibliometric studies have been conducted to investigate the top-cited papers in medical fields, such as diabetes, hypertension, osteoporosis, imaging, dentistry, cardiac surgery, and orthopaedics [4–10].

China is the most populous territory with over 1.3 billion people and is one of the most rapidly developing countries in the world [11]. Moreover, China has more than 50,000 orthopaedic surgeons [12]. The fracture research studies by these surgeons have resulted in great progress in recent years, gaining increasing attention from universities worldwide. China has an opportunity to become one of the leading forces in fracture surgery worldwide [13, 14]. However, to our knowledge, influential fracture papers that have originated in China have not been analysed. Therefore, the present study aimed to analyse the 50 top-cited fracture papers from China and to assess their characteristics to provide insight into Chinese research on fracture surgery.

## Methods

This study was conducted according to the Preferred Reporting Items for Systematic Reviews and Meta-Analyses statement [15]. We adopted the designs of previous publications as models for the design of this study [4–9]. On October 15, 2015, a citation search was conducted using the Web of Science (Thomson Reuters, Philadelphia, Pennsylvania, USA). A total of 72 journals were under the “Orthopedics” category of the Journal Citation Reports (JCR) for the year 2014. All of these journals were included in the search for the top-cited papers in this study.

The inclusion criteria were as follows: (1) fracture-related articles, (2) articles originating in China, and (3) those ranked as one of the top 50 papers according to the number of citations. Two authors independently conducted the literature search and data collection. Disagreements were resolved by discussion, and a third reviewer was consulted for the final decision if necessary. First, we entered the journal titles in the search window using the “OR” operator in the Web of Science database so that all articles from the 72 journals were identified. We then ranked the articles according to the number of citations. The search results were refined by selecting the filters “Peoples R China,” “Hong Kong,” “Taiwan,” and “Macau.” In addition, this study sought to include papers that orthopaedic trauma surgeons would find relevant to their practice. Thus, other articles on fractures, such as spine fracture, paediatric fracture, hand fracture distal to the wrist, stress fracture, infection not specifically related to fracture surgery, and arthroplasty, were excluded according to previous publications [16]. Each paper was reviewed, and papers without a first-author address from a Chinese region were excluded. Thereby, the inclusion of articles not originating in China but having one or more Chinese co-authors was avoided.

The 50 top-cited Chinese papers on fracture surgery were finally collected. The title, number of citations, year of publication, journal source, article type, level of evidence, city, institution, and authors were analysed. The level of evidence for clinical studies was in accordance with the guidelines published by *The Journal of Bone & Joint Surgery-American Volume* [17].

## Results

**Fig. 1:**
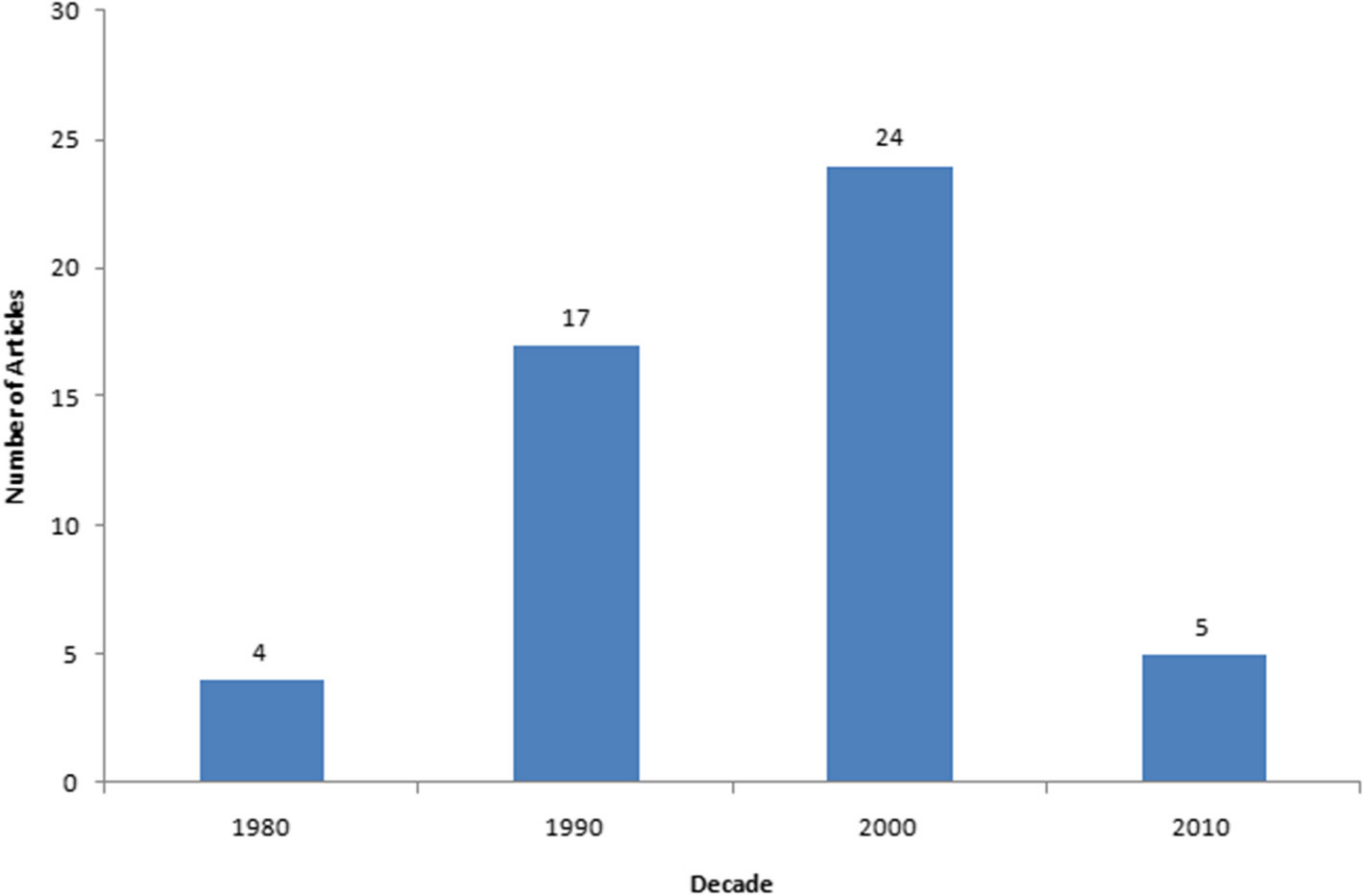
Number of articles by decade of publication

We initially identified 235,129 articles. The 50 most-cited articles from China were included in this study. Table 1 shows the 50 top-cited articles. The number of citations ranged from 209 to 28 (mean 52). The oldest paper, written in 1984, ranked 49th, while the most recent article, published in 2012, ranked 18th. The 2000s accounted for the most articles (24), followed by the 1990s (17), 2010s (5), and 1980s (4) (Fig. 1).

**Table 1:** The 50 most-cited articles in fracture surgery from China

Rank	Article	Number of citations
1	Wang SJ, Lewallen DG, Bolander ME, Chao EY, Ilstrup DM, Greenleaf JF. Low intensity ultrasound treatment increases strength in a rat femoral fracture model. J Orthop Res 1994;12(1):40–7.	209
2	Leung KS, So WS, Shen WY, Hui PW. Gamma nails and dynamic hip screws for peritrochanteric fractures. A randomised prospective study in elderly patients. J Bone Joint Surg Br 1992;74(3):345–51.	205
3	Wang CJ, Chen HS, Chen CE, Yang KD. Treatment of nonunions of long bone fractures with shock waves. Clin Orthop Relat Res 2001;387:95–101.	104
4	Fitoussi F, Ip WY, Chow SP. Treatment of displaced intra-articular fractures of the distal end of the radius with plates. J Bone Joint Surg Am 1997;79(9):1303–12.	84
5	Leung KS, Yuen KM, Chan WS. Operative treatment of displaced intra-articular fractures of the calcaneum. Medium-term results. J Bone Joint Surg Br 1993;75(2):196–201.	84
6	Wang FS, Yang KD, Chen RF, Wang CJ, Sheen-Chen SM. Extracorporeal shock wave promotes growth and differentiation of bone-marrow stromal cells towards osteoprogenitors associated with induction of TGF-beta1. J Bone Joint Surg Br 2002;84(3):457–61.	84
7	Chen YJ, Wurtz T, Wang CJ, Kuo YR, Yang KD, Huang HC, et al. Recruitment of mesenchymal stem cells and expression of TGF-beta 1 and VEGF in the early stage of shock wave-promoted bone regeneration of segmental defect in rats. J Orthop Res 2004;22(3):526–34.	79
8	Shen WJ, Liu TJ, Shen YS. Plate fixation of fresh displaced midshaft clavicle fractures. Injury 1999;30(7):497–500.	69
9	Leung KS, Cheung WH, Zhang C, Lee KM, Lo HK. Low intensity pulsed ultrasound stimulates osteogenic activity of human periosteal cells. Clin Orthop Relat Res 2004;418:253–9.	67
10	Leung F, Tu YK, Chew WY, Chow SP. Comparison of external and percutaneous pin fixation with plate fixation for intra-articular distal radial fractures. A randomized study. J Bone Joint Surg Am 2008;90(1):16–22.	63
11	Shen X, Wan C, Ramaswamy G, Mavalli M, Wang Y, Duvall CL, et al. Prolyl hydroxylase inhibitors increase neoangiogenesis and callus formation following femur fracture in mice. J Orthop Res 2009;27(10):1298–305.	63
12	Chiu KY, Pun WK, Luk KD, Chow SP. A prospective study on hip fractures in patients with previous cerebrovascular accidents. Injury 1992;23(5):297–9.	62
13	Leung KS, Shen WY, So WS, Mui LT, Grosse A. Interlocking intramedullary nailing for supracondylar and intercondylar fractures of the distal part of the femur. J Bone Joint Surg Am 1991;73(3):332–40.	62
14	Hung LK, Chan KM, Chow YN, Leung PC. Fractured patella: operative treatment using the tension band principle. Injury 1985;16(5):343–7.	58
15	Leung KS, Shen WY, Tsang HK, Chiu KH, Leung PC, Hung LK. An effective treatment of comminuted fractures of the distal radius. J Hand Surg Am 1990;15(1):11–7.	57
16	Lin J, Hou SM, Hang YS, Chao EY. Treatment of humeral shaft fractures by retrograde locked nailing. Clin Orthop Relat Res 1997;342:147–55.	56
17	Ko JY, Yamamoto R. Surgical treatment of complex fracture of the proximal humerus. Clin Orthop Relat Res 1996;327:225–37.	56
18	Hu F, Jiang C, Shen J, Tang P, Wang Y. Preoperative predictors for mortality following hip fracture surgery: a systematic review and meta-analysis. Injury 2012;43(6):676–85.	55
19	Wang CJ, Huang HY, Chen HH, Pai CH, Yang KD. Effect of shock wave therapy on acute fractures of the tibia: a study in a dog model. Clin Orthop Relat Res 2001;387:112–8.	52
20	Wang Q, Zhong S, Ouyang J, Jiang L, Zhang Z, Xie Y, et al. Osteogenesis of electrically stimulated bone cells mediated in part by calcium ions. Clin Orthop Relat Res 1998;348:259–68.	50
21	Leung KS, Lam TP. Open reduction and internal fixation of ipsilateral fractures of the scapular neck and clavicle. J Bone Joint Surg Am 1993;75(7):1015–8.	46
22	Leung KS, Shen WY, Leung PC, Kinninmonth AW, Chang JC, Chan GP. Ligamentotaxis and bone grafting for comminuted fractures of the distal radius. J Bone Joint Surg Br 1989;71(5):838–42.	45
23	Yang RS, Tsuang YH, Hang YS, Liu TK. Traumatic dislocation of the hip. Clin Orthop Relat Res 1991;265:218–27.	40
24	Lau TW, Leung F, Chan CF, Chow SP. Wound complication of minimally invasive plate osteosynthesis in distal tibia fractures. Int Orthop 2008;32(5):697–703.	40
25	Wang JW, Weng LH. Treatment of distal femoral nonunion with internal fixation, cortical allograft struts, and autogenous bone-grafting. J Bone Joint Surg Am 2003;85-A(3):436–40.	39
26	Zhiquan A, Bingfang Z, Yeming W, Chi Z, Peiyan H. Minimally invasive plating osteosynthesis (MIPO) of middle and distal third humeral shaft fractures. J Orthop Trauma 2007;21(9):628–33.	39
27	Leung F, Chow SP. A prospective, randomized trial comparing the limited contact dynamic compression plate with the point contact fixator for forearm fractures. J Bone Joint Surg Am 2003;85-A(12):2343–8.	36
28	Luo CF, Sun H, Zhang B, Zeng BF. Three-column fixation for complex tibial plateau fractures. J Orthop Trauma 2010;24(11):683–92.	36
29	Lin J, Hou SM. Antegrade locked nailing for humeral shaft fractures. Clin Orthop Relat Res 1999;365:201–10.	35
30	Leung KS, Shi HF, Cheung WH, Qin L, Ng WK, Tam KF, et al. Low-magnitude high-frequency vibration accelerates callus formation, mineralization, and fracture healing in rats. J Orthop Res 2009;27(4):458–65.	34
31	Mak KH, Chan KM, Leung PC. Ankle fracture treated with the AO principle—an experience with 116 cases. Injury 1985;16(4):265–72.	33
32	Cheung KM, Kaluarachi K, Andrew G, Lu W, Chan D, Cheah KS. An externally fixed femoral fracture model for mice. J Orthop Res 2003;21(4):685–90.	33
33	Xu XL, Tang T, Dai K, Zhu Z, Guo XE, Yu C, et al. Immune response and effect of adenovirus-mediated human BMP-2 gene transfer on the repair of segmental tibial bone defects in goats. Acta Orthop 2005;76(5):637–46.	32
34	Liu Y, Tao R, Liu F, Wang Y, Zhou Z, Cao Y, et al. Mid-term outcomes after intramedullary fixation of peritrochanteric femoral fractures using the new proximal femoral nail antirotation (PFNA). Injury 2010;41(8):810–7.	32
35	Fan CY, Chiang CC, Chuang TY, Chiu FY, Chen TH. Interlocking nails for displaced metaphyseal fractures of the distal tibia. Injury 2005;36(5):669–74.	32
36	Huang HT, Huang PJ, Su JY, Lin SY. Indirect reduction and bridge plating of supracondylar fractures of the femur. Injury 2003;34(2):135–40.	31
37	Dai KR, Hou XK, Sun YH, Tang RG, Qiu SJ, Ni C. Treatment of intra-articular fractures with shape memory compression staples. Injury 1993;24(10):651–5.	31
38	Hsueh KK, Fang CK, Chen CM, Su YP, Wu HF, Chiu FY. Risk factors in cutout of sliding hip screw in intertrochanteric fractures: an evaluation of 937 patients. Int Orthop 2010;34(8):1273–6.	31
39	Guo JJ, Tang N, Yang HL, Tang TS. A prospective, randomised trial comparing closed intramedullary nailing with percutaneous plating in the treatment of distal metaphyseal fractures of the tibia. J Bone Joint Surg Br 2010;92(7):984–8.	31
40	Hung LK, Wu HT, Leung PC, Qin L. Low BMD is a risk factor for low-energy Colles’ fractures in women before and after menopause. Clin Orthop Relat Res 2005;435:219–25.	30
41	Leung KS, Chen CM, So WS, Sato K, Lai CH, Machaisavariya B, et al. Multicenter trial of modified Gamma nail in East Asia. Clin Orthop Relat Res 1996;323:146–54.	30
42	Pu JS, Liu L, Wang GL, Fang Y, Yang TF. Results of the proximal femoral nail anti-rotation (PFNA) in elderly Chinese patients. Int Orthop 2009;33(5):1441–4.	30
43	Wong MK, Leung F, Chow SP. Treatment of distal femoral fractures in the elderly using a less-invasive plating technique. Int Orthop 2005;29(2):117–20.	30
44	Jiang R, Luo CF, Zeng BF, Mei GH. Minimally invasive plating for complex humeral shaft fractures. Arch Orthop Trauma Surg 2007;127(7):531–5.	29
45	Wu CC, Shih CH. Biomechanical analysis of the mechanism of interlocking nail failure. Arch Orthop Trauma Surg 1992;111(5):268–72.	29
46	Tao J, Hang DH, Wang QG, Gao W, Zhu LB, Wu XF, et al. The posterolateral shearing tibial plateau fracture: treatment and results via a modified posterolateral approach. Knee 2008;15(6):473–9.	29
47	Lee YS, Lin CC, Huang CR, Chen CN, Liao WY. Operative treatment of midclavicular fractures in 62 elderly patients: knowles pin versus plate. Orthopedics 2007;30(11):959–64.	29
48	Yang SW, Tzeng HM, Chou YJ, Teng HP, Liu HH, Wong CY. Treatment of distal tibial metaphyseal fractures: plating versus shortened intramedullary nailing. Injury 2006;37(6):531–5.	28
49	Chan KM, Leung YK, Cheng JC, Leung PC. The management of type III open tibial fractures. Injury 1984;16(3):157–65.	28
50	Jiang R, Luo CF, Wang MC, Yang TY, Zeng BF. A comparative study of less invasive stabilization system (LISS) fixation and two-incision double plating for the treatment of bicondylar tibial plateau fractures. Knee 2008;15(2):139–143.	28

The 50 top-cited articles were published in English in 12 journals (Table 2). *Injury* (11) published the largest number of papers, followed by *Clinical Orthopaedics and Related Research* (10), *The Journal of Bone & Joint Surgery-American Volume* (6), *The Journal of Bone & Joint Surgery-British Volume* (5), and *Journal of Orthopaedic Research* (5).

**Table 2:** Number of articles by source journal

Journal	Number of articles	Impact factor
*Injury*	11	2.137
*Clinical Orthopaedics and Related Research*	10	2.765
*The Journal of Bone & Joint Surgery American Volume*	6	5.28
*The Journal of Bone & Joint Surgery British Volume*	5	3.309
*Journal of Orthopaedic Research*	5	2.986
*International Orthopaedics*	4	2.11
*Archives of Orthopaedic and Trauma Surgery*	2	1.597
*Journal of Orthopaedic Trauma*	2	1.803
*The Knee*	2	1.936
*The Journal of Hand Surgery*	1	1.667
*Acta Orthopaedica*	1	2.771
*Orthopedics*	1	0.962

**Fig. 2:**
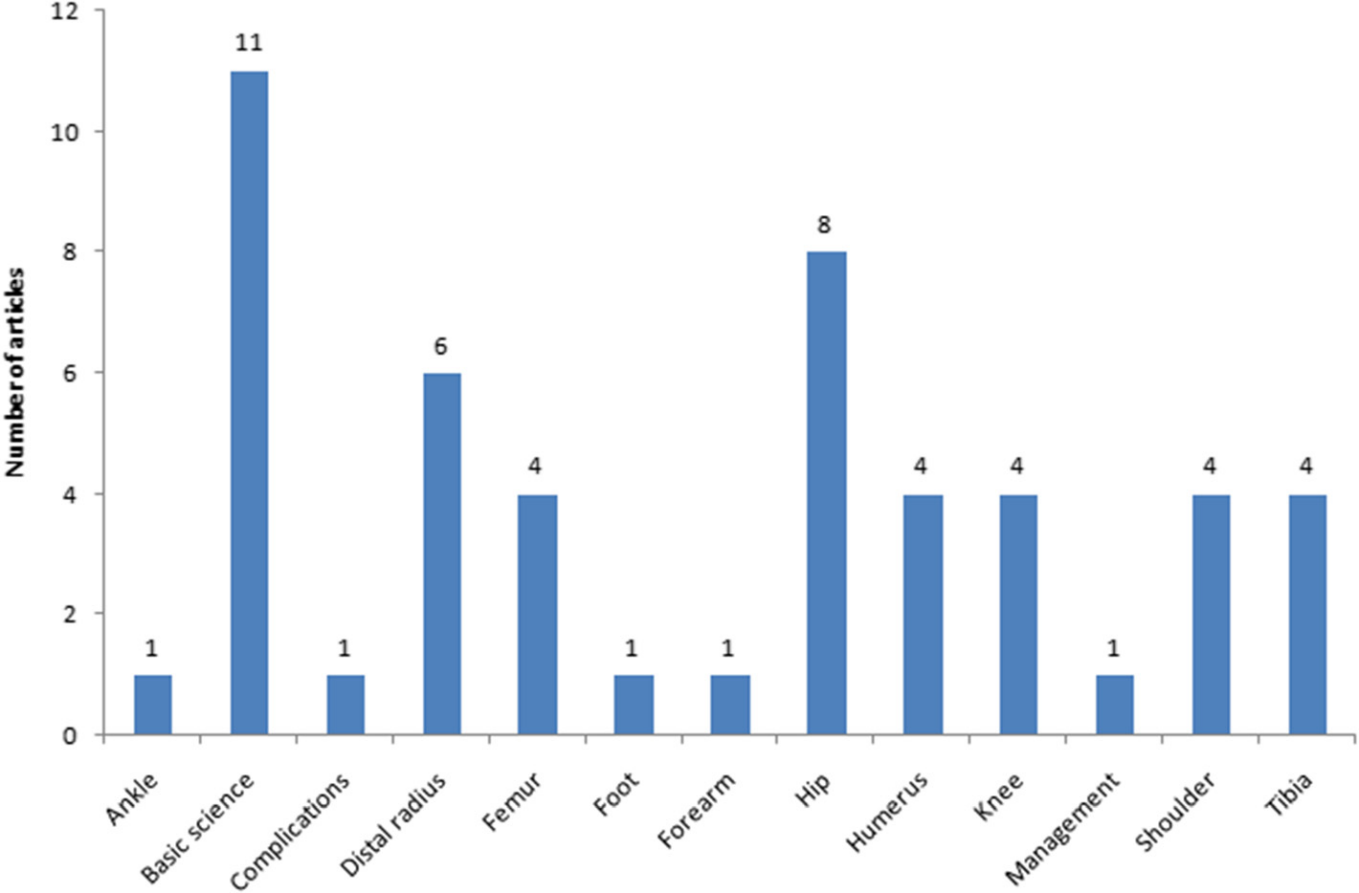
Classification of articles by topic

**Fig. 3:**
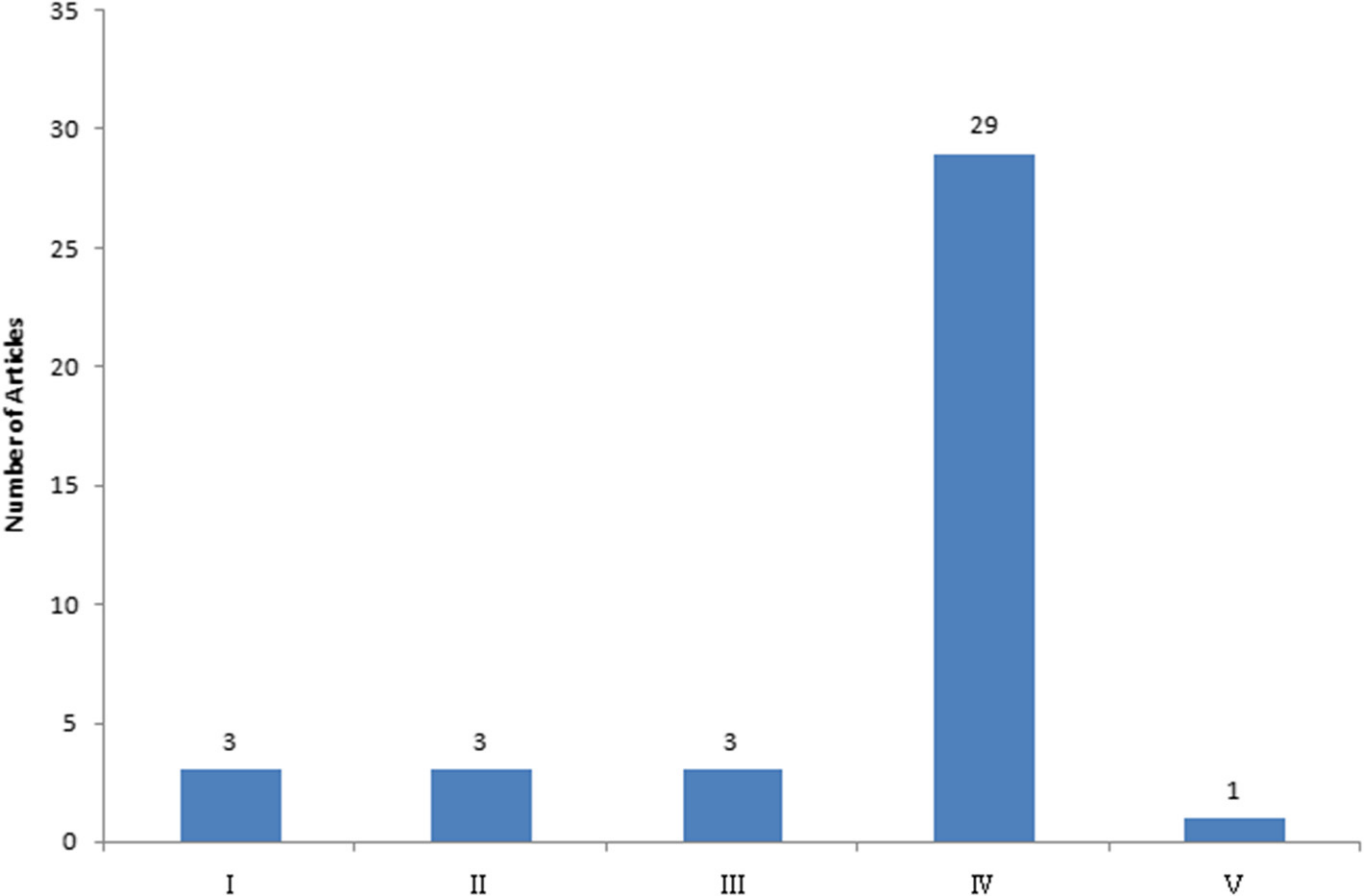
Number of clinical articles by level of evidence

Eleven studies were basic science articles, while 39 were clinical studies. The hip was the most discussed topic in the clinical studies, followed by the distal radius (Fig. 2). The most common article type among the clinical studies was case series (Table 3). Most of the clinical studies had a level of evidence of IV (29) (Fig. 3).

**Table 3:** Clinical articles classified by study type

Study type	Number of articles
Randomised controlled trial	6
Cohort study	3
Case series	29
Review article	1

The top 50 papers originated from mainland China (13), Hong Kong (20), and Taiwan (17). None of the articles originated from Macau (0). Eleven cities contributed to these 50 articles, led by Hong Kong (20) and followed by Kaohsiung (8), Shanghai (8), and Taipei (7) (Table 4). Twenty-two institutions contributed to the top 50 articles. Eight institutions had at least two articles published. The Prince of Wales Hospital had the most published papers (11), followed by Queen Mary Hospital (6) and Chang Gung Memorial Hospital in Kaohsiung (5) (Table 5). Multiple first-authors appeared more than once in the top 50 articles. The top first author was Leung KS (9), followed by Wang CJ (2), Leung F (2), and Lin J (2) (Table 6).

**Table 4:** Cities of origin of articles

City	Number of articles
Hong Kong	20
Kaohsiung	8
Shanghai	8
Taipei	7
Taoyuan	2
Guangzhou	1
Chengdu	1
Soochow	1
Nantong	1
Tianjin	1

**Table 5:** Institutions with more than one article

Institution (city)	Number of articles
Prince of Wales Hospital (Hong Kong)	11
Queen Mary Hospital (Hong Kong)	6
Chang Gung Memorial Hospital (Kaohsiung)	5
National Taiwan University Hospital (Taipei)	3
Princess Margaret Hospital (Hong Kong)	3
Shanghai Sixth People’s Hospital (Shanghai)	3
Chang Gung Memorial Hospital (Taoyuan)	2
Ninth People’s Hospital (Shanghai)	2
Veterans General Hospital (Taipei)	2

**Table 6:** Authors who were first-authors in more than one article

First author	Number of articles
Leung KS	9
Wang CJ	2
Leung F	2
Lin J	2

## Discussion

The top-cited articles represent the most influential articles in their respective fields [4–9]. Citation analysis helps to identify significant research studies, the important contributors, and their main characteristics [4–9, 16, 18]. In addition, understanding the historical context, developing processes, and future trends in a certain field can be helpful [4–9, 18]. The number of publications regarding investigations of the most-cited articles in medical fields has been increasing [4–9]. Similarly, a paper that investigated the most-cited articles on fracture surgery was published in 2013 [16]. However, only one Chinese article was included in that study [16]. Therefore, the most-cited articles from Chinese authors in the field of fracture had not been investigated despite the increasing importance of Chinese research studies in the global community. To our knowledge, this is the first study to have identified the top-cited Chinese articles on fracture surgery.

The number of citations of the 50 top-cited publications ranged from 209 to 28. The reason that few Chinese articles have appeared on previous top article lists may be due to the smaller number of citations than those in the global community [16]. This result indicates that even though there has been a rapid increase in research output, Chinese authors should also work to improve the quality of their research studies [19]. However, this study also demonstrates that the 2000s was the most productive decade, indicating an increasing volume of important research studies from Chinese authors. It is reasonable to expect that China will contribute more influential papers in the future.

The 50 papers were all published in English, probably because English is the most common language used in fracture surgery articles [16]. Moreover, this may suggest a reason why fewer fracture articles from China were published in English journals, as Chinese authors may find it difficult to write their research studies in English.

The top 50 papers were published in 12 journals. The journal that included the largest number of papers was *Injury*, indicating that high-level Chinese articles on fracture surgery are likely to be published in *Injury*. Therefore, this journal plays an important role in the sharing of Chinese knowledge in the field of fracture surger*y.* A previous study demonstrated that the impact factor of the journal was the most important indicator of the number of citations, and a large proportion of the most-cited papers were published in other high-impact journals [20]. However, this was not borne out by our study. *The Journal of Bone & Joint Surgery-American Volume*, which has the highest impact factor (5.28) on the journal list, published fewer articles than *Injury*. This result suggests that citations are not necessarily affected by the journal impact factor because various other factors contributed to the citations for certain journals [10, 21].

Although mainland China has the highest number of patients and physicians among the Chinese regions, Hong Kong published the most papers on the top 50 list. This indicates that Hong Kong produces more high-quality papers than other Chinese regions. Similar findings have also been published previously [22]. Eleven cities were responsible for the top 50 papers. The ranking was led by Hong Kong, followed by Kaohsiung, Shanghai, and Taipei. These top four cities published nearly 90 % of the articles on the top 50 list. This finding confirms the important influence of these cities on fracture surgery in China. It also suggests that high-level Chinese research on fracture surgery is concentrated in several cities, which can be attributed to the large size of the orthopaedic communities in these cities and sufficient financial support [23, 24].

On the top 50 list, clinical studies were more popular than basic science research, which is consistent with the situation in the global community [16]. One of the contributing factors may be that China’s large population is advantageous to the recruitment of participants. The clinical articles focused on a variety of different topics. Hip fracture was the most discussed topic, which was consistent with the findings of a previous publication [16]. Most clinical articles had level IV evidence. This result indicates that the level of evidence do not necessarily influence the total number of citations. Moreover, some institutions and authors had good publication records in the field of fracture surgery; for example, The Prince of Wales Hospital and Leung KS were at the top of the list. Their output of high-level articles suggests their specific contributions to the field of fracture surgery and their great impact in China.

This study has several limitations. First, high-impact articles were identified through a validated method, but influential Chinese papers with fewer citations might have been excluded. Second, we used categories to identify orthopaedic journals, which might not include all fracture articles. Influential Chinese articles on general medicine and basic research journals were not evaluated. Third, multiple factors influenced the number of citations. Self-citation and citations in textbooks and lectures could not be investigated in this study [10, 25, 26]. In addition, the authors tended to cite papers from the journals to which they submit their own research studies [27]. Fourth, this is a study with a cross-sectional design conducted at a single time point. The rankings of the list could change if the study were to be performed at a subsequent time point.

## Conclusions

This study is the first bibliometric study of the most-cited Chinese papers on fracture surgery. The present study shows the most influential Chinese articles for global orthopaedic trauma surgeons and provides an overview of the impact of Chinese research studies on fracture surgery. This study provides historical insight into Chinese fracture research and the trends in the field of fracture surgery in China.

## Abbreviations

JCR, Journal Citation Reports

## References

[1] Garfield E (1972). Citation analysis as a tool in journal evaluation. Science.

[2] Cheek J, Garnham B, Quan J (2006). What’s in a number? Issues in providing evidence of impact and quality of research(ers). Qual Health Res.

[3] Gisvold SE (1999). Citation analysis and journal impact factors—is the tail wagging the dog?. Acta Anaesthesiol Scand.

[4] Shuaib W, Costa JL (2015). Anatomy of success: 100 most cited articles in diabetes research. Ther Adv Endocrinol Metab.

[5] Oh YS, Galis ZS (2014). Anatomy of success: the top 100 cited scientific reports focused on hypertension research. Hypertension.

[6] Holzer LA, Leithner A, Holzer G (2015). The most cited papers in osteoporosis and related research. J Osteoporos.

[7] Brinjikji W, Klunder A, Kallmes DF (2013). The 100 most-cited articles in the imaging literature. Radiology.

[8] Feijoo JF, Limeres J, Fernandez-Varela M, Ramos I, Diz P (2014). The 100 most cited articles in dentistry. Clin Oral Investig.

[9] O’Sullivan KE, Kelly JC, Hurley JP (2015). The 100 most cited publications in cardiac surgery: a bibliometric analysis. Ir J Med Sci.

[10] Lefaivre KA, Shadgan B, O’Brien PJ (2011). 100 most cited articles in orthopaedic surgery. Clin Orthop Relat Res.

[11] China. The World Bank. Available from: http://data.worldbank.org/country/china.

[12] Leung KS, Ngai WK, Tian W (2011). Orthopaedic training in China: experiences from the promotion of orthopaedic specialist training in China. J Bone Joint Surg Br.

[13] Perkovic V, Patil V, Wei L, Lv J, Petersen M, Patel A (2012). Global randomized trials: the promise of India and China. J Bone Joint Surg Am.

[14] Wang C, Liu Q (2013). A turning point for clinical research in China?. Lancet.

[15] Liberati A, Altman DG, Tetzlaff J, Mulrow C, Gotzsche PC, Ioannidis JP (2009). The PRISMA statement for reporting systematic reviews and meta-analyses of studies that evaluate health care interventions: explanation and elaboration. PLoS Med.

[16] Baldwin K, Namdari S, Donegan D, Kovatch K, Ahn J, Mehta S (2013). 100 most cited articles in fracture surgery. Am J Orthop (Belle Mead NJ).

[17] Wright JG, Swiontkowski MF, Heckman JD (2003). Introducing levels of evidence to the journal. J Bone Joint Surg Am.

[18] Huo YQ, Pan XH, Li QB, Wang XQ, Jiao XJ, Jia ZW (2015). Fifty top-cited classic papers in orthopedic elbow surgery: a bibliometric analysis. Int J Surg.

[19] Cheng T (2012). Research in orthopaedics from China has thrived over the last decade: a bibliometric analysis of publication activity. Orthop Traumatol Surg Res.

[20] Callaham M, Wears RL, Weber E (2002). Journal prestige, publication bias, and other characteristics associated with citation of published studies in peer-reviewed journals. JAMA.

[21] Murray MR, Wang T, Schroeder GD, Hsu WK (2012). The 100 most cited spine articles. Eur Spine J.

[22] Jia ZW, Wu YH, Li H, Li HF, Zhao XY, Tang Y (2015). Growing trend of China’s contribution to the field of spine: a 10-year survey of the literature. Eur Spine J.

[23] Pagni M, Khan NR, Cohen HL, Choudhri AF (2014). Highly cited works in radiology: the top 100 cited articles in radiologic journals. Acad Radiol.

[24] Kelly JC, Glynn RW, O’Briain DE, Felle P, McCabe JP (2010). The 100 classic papers of orthopaedic surgery: a bibliometric analysis. J Bone Joint Surg Br.

[25] Namdari S, Baldwin K, Kovatch K, Huffman GR, Glaser D (2012). Fifty most cited articles in orthopedic shoulder surgery. J Shoulder Elbow Surg.

[26] Dumont JE (1989). The bias of citations. Trends Biochem Sci.

[27] Seglen PO (1997). Why the impact factor of journals should not be used for evaluating research. BMJ.

